# Telomere maintenance in soft tissue sarcomas

**DOI:** 10.1136/jclinpath-2016-204151

**Published:** 2017-04-18

**Authors:** Nicholas Eastley, Barbara Ottolini, Carmen Garrido, Jacqueline A Shaw, Thomas Alasdair McCulloch, Robert U Ashford, Nicola J Royle

**Affiliations:** 1 Department of Genetics, University of Leicester, Leicester, UK; 2 Department of Cancer Studies, University of Leicester, Leicester, UK; 3 Department of Histopathology, Nottingham University Hospitals NHS Trust, Nottingham, UK; 4 University Hospitals of Leicester NHS Trust, Leicester, UK

**Keywords:** SARCOMAS, CANCER GENETICS, GENETICS, SOFT TISSUE TUMOURS

## Abstract

Soft tissue sarcomas (STS) are a diverse group of heterogeneous malignant tumours derived from mesenchymal tissues. Over 50 different STS subtypes are recognised by WHO, which show a wide range of different biological behaviours and prognoses. At present, clinicians managing this complex group of tumours face several challenges. This is reflected by the relatively poor outcome of patients with STSs compared with many other solid malignant tumours. These include difficulties securing accurate diagnoses, a lack of effective systemic treatments and absence of any sensitive circulating biomarkers to monitor patients throughout their treatment and follow-up. In order to progress STS's cells must evade the usual cellular proliferative checkpoints, and then activate a telomere maintenance mechanism in order to achieve replicative immortality. The purpose of this review is to provide an overview of STS genetics focusing particularly on these mechanisms. We will also highlight some of the key barriers to improving outcome for patients with STS, and hypothesise how a better understanding of these genetic characteristics may impact on future STS management.

Soft tissue sarcomas (STSs) are rare malignant solid tumours derived from mesenchymal tissues. Over 50 different STS subtypes have been classified by WHO, many of which have profound differences in their genetic makeup, histological characteristics, biological behaviour and prognoses. The outcome for patients with STSs is relatively poor compared with many other solid malignant tumours. Clinicians face several challenges when managing STSs including delays to presentation, difficulties securing diagnoses, limited systemic treatment options and a lack of effective tools to monitor patients throughout their treatment and follow-up. The purpose of this review is to provide an overview of STS genetics focusing particularly on telomere maintenance (a necessity for malignant cells in order to achieve immortality). We will also highlight some of the key barriers to improving outcome for patients with STS, and hypothesise how a better understanding of these genetic characteristics may impact on future STS management.

## Telomeres

### Telomere function

In eukaryotic cells DNA is packaged into linear chromosomes. Although this facilitates the maintenance of genetic variability through recombination and random chromosomal assortment during meiosis, storage of DNA in this way has two disadvantages. First, the inability of DNA polymerase to replicate the very terminal ends of a linear chromosome during mitosis (a problem termed the ‘end replication problem’^1^) means linear chromosomes progressively shorten during successive cell divisions, leaving them susceptible to degradation over time. Second, if the ends of linear chromosomes are not protected they run the risk of triggering a cell's double-strand DNA damage response (DDR) pathway, which in turn would result in abnormal chromosomal end-to-end fusions and genome instability.

To overcome these disadvantages, DNA-protein complexes called telomeres exist at the ends of eukaryotic chromosomes. Telomeres maintain genome stability by shielding the terminal coding sequences of chromosomes from degradation during cellular replication, and also by differentiating chromosome ends from abnormal chromosome breakages preventing activation of the DDR pathway. A further function of telomeres is to provide protection against the development of cancer by acting as an internal lifespan for the cells they are found in. After a certain number of divisions (known as a cell's ‘Hayflick limit’^2^), telomere shortening (predominantly due to the end replication problem) triggers a cell to enter replicative senescence and undergo growth arrest. Although cells in this senescent state remain metabolically active, they cease to divide, preventing further DNA erosion, genomic instability and the accumulation of potentially oncogenic mutations.

### Telomere structure

**Figure 1:**
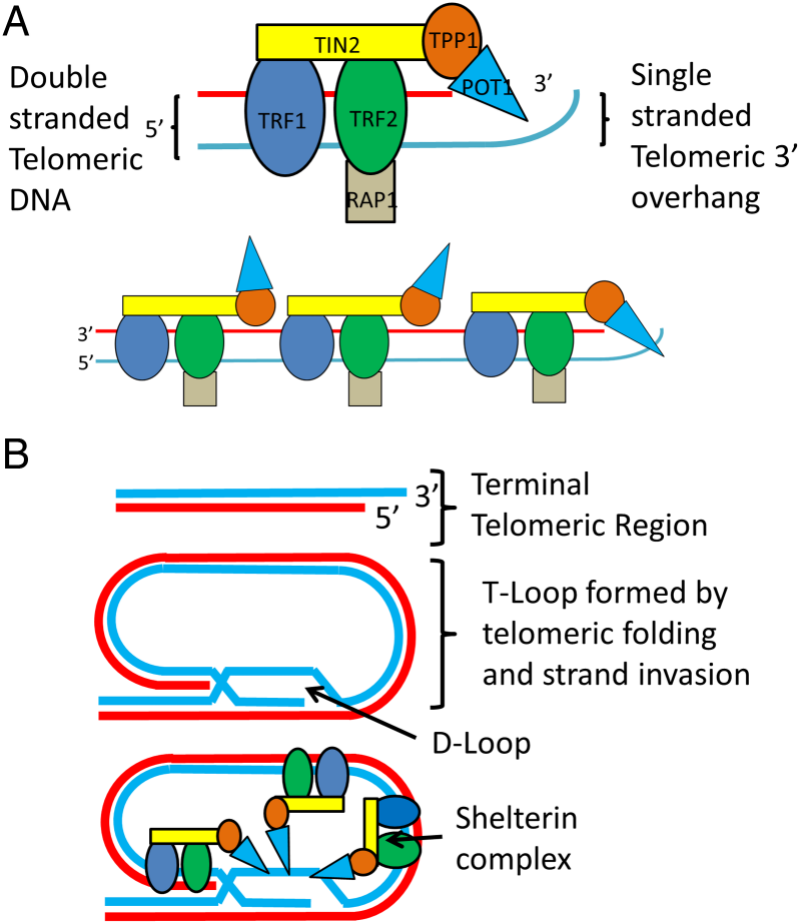
The shelterin protein complex structure and interactions with telomeric DNA. (A) shows the secondary structure of the six protein subunits that make up the shelterin complex. Rap1 binds to TRF2, TPP1 binds POT1, and TIN2 binds TRF1, TRF2 and TPP1. TRF1 and TRF2 bind to double-stranded telomeric DNA, while POT1 binds to single-stranded telomeric DNA. The relationship between multiple shelterin complexes and the terminal region of a linear, unlooped telomere is also shown. Shelterin regulates the length of linear telomeres through the binding of POT1 to the single-stranded telomeric overhang region which blocks telomerase binding. (B) shows a telomere's single-stranded 3′ overhang folding back and self-invading an adjacent canonical repeat region forming D-loop and T-loop structures (a process mediated by shelterin). Throughout the cell cycle telomeres switch dynamically between this looped conformation and the linear structure in (A). T-loops protect telomeres by preventing activation of the double-stranded break repair mechanisms and also regulate telomere length by preventing telomerase binding to telomeres. Multiple shelterin complexes are also shown interacting with a T-loop.

The DNA component of human telomeres consists of simple tandem repeat arrays (STRs) of TTAGGG (the canonical telomeric sequence) that equates to 2000–15 000 base pairs (bp). Almost all of this DNA is double stranded other than a short (<300 bp) single-stranded extension found at the very terminal end of the 3′ G-rich end called the 3′ overhang.^3^ Throughout the cell cycle telomeres shift between a linear and looped configuration created when 3′ G-rich overhangs fold back and hybridise within their own double-stranded portions. In both conformations, several proteins protect telomeres by preventing the activation of the DDR repair mechanism, and regulate telomere length by inhibiting the enzyme telomerase. One of the most important of these protein complexes is called shelterin, which binds to telomeric DNA primarily through two of its components called TRF1 and TRF2^4^ (see figure 1 for more detail).

### Telomere maintenance and cancer

Several models have been proposed to explain how solid malignant tumours grow, regenerate and show such high levels of intratumour heterogeneity. One example is the ‘cancer stem model’, which proposes that certain tumour cells (cancer stem cells) are able to differentiate into malignant cells of a distinct type.^5^ Another model is the ‘clonal evolution model’, which instead hypothesises that genomic instability (and the resulting accumulation of somatic oncogenic mutations) creates subsets of tumour cells with a proliferative advantage and metastatic potential.

**Figure 2:**
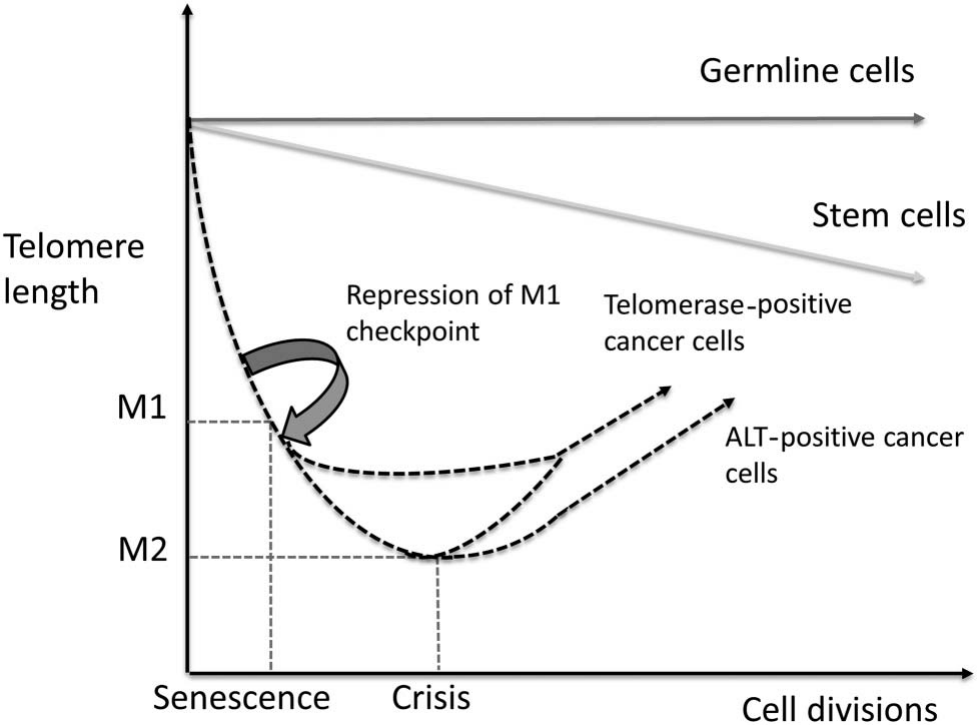
Graphic representation of telomere shortening with repeated cell divisions in germ line cells, stem cells, telomerase positive and ALT positive cancer cells with incompetent M1 checkpoints. As shown telomerase activation may occur at any point following evasion of the M1 checkpoint whereas ALT is most likely initiated at M2 (cellular crisis).

When healthy cells reach their Hayflick limit, tumour suppressor pathways involving tumour protein p53 and retinoblastoma protein (pRb) trigger replicative senescence (see figure 2). This checkpoint is known as mortality stage 1 (M1) and functions to prevent uncontrolled cell division. If a cell acquires somatic mutations that inhibit p53 or pRB it will bypass M1. When this occurs mitosis and telomere shortening continues until a second proliferative checkpoint called M2 (or cellular ‘crisis’) is entered. This checkpoint is characterised by severe genomic instability (driven by telomere fusions and breakages) and widespread cell death. Regardless of which model underpins tumour progression, in order to survive a tumour's malignant cells must evade both M1 and M2 checkpoints.^6^ Following this they must also activate a mechanism to maintain or lengthen their telomeres^7^ in order to avoid senescence and achieve replicative immortality. This ability is facilitated by one of two ‘telomere maintenance mechanisms (TMMs)’—the action of the enzyme telomerase or the alternative lengthening of telomeres (ALT).

## Telomerase

### Structure and function

Telomerase is a reverse transcriptase enzyme with two main functional units—human telomerase RNA (hTR) and human telomerase reverse transcriptase (hTERT). Together with several other accessory proteins including dyskerin, NHP2, TCAB1, NOP10 and GAR1, hTR and hTERT act together to mediate telomere lengthening in the majority of solid tumours.^8^

After telomerase assembly in the Cajal body (a process promoted by TCAB1), the enzyme is transported to telomeres by shelterin's POT1-TPP1 complex. hTR is a RNA that contains an 11 nucleotide template complementary to the canonical telomeric sequence. Following telomeric T-loop dissociation and telomere processing by the CST (CTC1, STN1 and TEN1) complex and Apollo, hTR initialises telomere lengthening by partially hybridising with telomere 3′ overhangs. Next hTERT (a catalytic protein subunit) elongates the involved telomere by using hTR as a template, before the detaching and translocating to the newly synthesised DNA 3′ end of the telomere so that the process can be repeated. Telomerase only lengthens one strand of a telomere. To complete the process of lengthening a DNA primase synthesises an RNA primer when telomerase detaches from a telomere. Using this primer, DNA polymerase α (the enzyme responsible for initiating DNA replication at origins of replication) generates a DNA strand complementary to the newly synthesised single-stranded 3′ overhang (the C strand). Finally, following adequate telomere lengthening the CST complex is again involved by rendering the lengthened telomere ‘closed’ to further telomerase-mediated extension.^8^ ^9^

### Telomerase regulation and hTERT promoter mutations

With the exception of fetal development, in germline cells and in certain cells with a high proliferative rate telomerase is generally inactive in healthy somatic cells. In contrast, the enzyme is commonly expressed in solid malignant tumours, and maintains telomere length in 80%–90% of human cancers overall.^10^ ^11^ Using PCR-based assays including the well-established telomeric repeat amplification protocol (TRAP) assay, a hugely varied incidence of telomerase activity has been reported in STSs (9%–81%).^12–17^

Telomerase may be regulated in several ways, including via assembly of the enzyme's components or delivery of the enzyme itself to telomeres. Primarily however the enzyme is controlled by the gene that encodes for hTERT—*TERT.* The promoter region of *TERT* contains binding sites for several transcription factors, which facilitate telomerase upregulation or downregulation by oncogenes or cancer suppressors, respectively.^18^ One mode of significant telomerase upregulation follows the acquisition of somatic mutations within the *TERT* promoter region. These create novel binding sites for transcription factors,^19^ and have been identified in many cancers including melanoma (71%),^20^ glioblastomas (100%),^21^ adrenocortical carcinomas (12%),^22^ small cell carcinoma of the bladder (100%)^23^ and thyroid cancers (8%–33%).^24^ The two most common *TERT* promoter alterations are single nucleotide base changes (cytosine to thymine transitions) at positions chr5:1,295,228 (C228T) and chr5:1,295,250 (C250T). Although certain STS subtypes commonly possess these mutations (eg, myxoid liposarcomas), they appear to be relatively rare in STSs overall, with a combined incidence of just 11%.^25^ ^26^ Interestingly, there also appears to be a relationship between ethnicity and the presence of *TERT* promoter mutations, which is yet to be explained.^27^

## The ALT mechanism

The observation that some dividing cells can maintain telomere length in the absence of telomerase led to the discovery of a telomerase-independent TMM called the ALT mechanism.^28^ ^29^ In contrast to telomerase activation, telomere lengthening by ALT most likely arises in cells at crisis, as extraordinary genome instability is a feature of ALT-positive tumours. The rapid lengthening and shortening of telomere length in telomerase-negative immortal cancer cells^30^ suggests that ALT maintains telomere length using a DNA recombination-mediated mechanism. This assumption forms the basis of the ‘homologous recombination-dependent DNA replication model’ for ALT. The repetitive nature of telomeric DNA means that canonical repeats may act as copy templates during this process, with one telomere molecule acting as a donor and another the recipient. This telomeric DNA copying may occur in one of several ways. First, strand invasion and copying may occur between sister-telomeres or between telomeres on different chromosomes.^31^ ^32^ Second, a telomere may loop back and invade its own telomeric region (forming a T-loop) providing its own copy template.^33^ Third, linear or circular extrachromosomal telomeric DNA may provide the required template. In this scenario, linear DNA may strand invade the lengthening telomere in a similar way to a sister chromatid, while circular DNA (t-circles or c-circles) may facilitate lengthening by a process termed rolling circle replication.^34^

### ALT and cancer

ALT-positive cells have several characteristic phenotypes. First, their telomeres are more heterogeneous in terms of length than those found in telomerase-positive cells,^29^ suggesting rapid telomeric shortening and lengthening between cell divisions. Second, ALT-positive cells contain higher levels of extrachromosomal telomeric DNA than telomerase-positive or healthy cells.^35^ Third, in ALT-positive cells a proportion of chromosomal and extrachromosomal telomeric DNA colocalises with telomere-binding proteins and promyelocytic leukaemia protein (PML) to form structures called ‘ALT-associated PML bodies’ (APBs)^36^. Finally, in ALT-positive cells sister chromatid and interchromosomal telomere exchanges are significantly more common than in telomerase-positive cells or healthy human cells.^31^ ^37^

By identifying these phenotypic features ALT has been identified in many malignant tumours. Although ALT is rarely seen in carcinomas (breast 4%, uterine 1%, oesophageal 1%, biliary 2%, renal 10%, hepatic tumours 8%), it is more common in malignant bladder tumours (28%), neurological tumours including glioblastoma multiforme (25%), germ cell tumours (15%) and sarcomas.^38–40^ Here, the presence of ALT varies significantly between different STS subtypes, with phenotypes previously reported in 63% of undifferentiated pleomorphic sarcomas, 53% of leiomyosarcomas, 33% of epithelioid sarcomas, 24%–26% of liposarcomas overall^17^ ^36^ ^41^ (0% well-differentiated, 30% dedifferentiated, 5% myxoid or round cell and 80% pleomorphic liposarcomas),^42^ 14% of fibrosarcomas and 11%–28% of angiosarcomas.^40^ ^43^ It has also been noted that the presence of ALT consistently correlates with higher levels of genomic instability, aggressive histological features and a worse prognosis in certain liposarcomas^17^ and leiomyosarcomas.^44^

## Clinical potential of TMMs

### Pathological assessment of STSs

Unfortunately in the UK, the average STS is over 10 cm in diameter at diagnosis^45^ (larger than many other solid malignant tumours) and a significant proportion of patients possess metastatic disease when they obtain a histological diagnosis. According to the American Joint Committee on Cancer/International Union against Cancer staging system, both of these characteristics are unsurprisingly poor prognostic indicators.

Retrospectively, a significant period of time can often be identified between the onset of a STS's symptoms and the time a patient is reviewed in an appropriate tertiary setting (median 14 months^46^). Efforts are being made to address this by raising STS awareness among both the public and medical professionals operating outside the sarcoma field.^47^ Despite this, these delays clearly highlight the importance of a rapid, accurate histological diagnosis being made once a biopsy is performed.

The histopathological diagnosis of a STS initially involves an assessment of cell morphology. Based on this appearance, STSs are broadly categorised as spindle-cell, small round cell, pleomorphic or epithelioid in nature. Although many STSs differentiate towards a specific phenotype, microscopic similarities between their morphology and cellular pleomorphism means this assessment alone is often inadequate to differentiate between subtypes, particularly based on a core biopsy alone.

STS subtypes can be categorised into two groups according to the somatic genetic alterations (including mutations and copy number aberrations) they possess. Although the exact consequence of many of these alterations is unknown, they are likely to play a critical role in cell cycle disruption and tumourigenesis. The first group contains around 20% of STSs that possess relatively simple alterations and near diploid karyotypes. The majority of mutations in this group are either reciprocal translocations that result in novel chimeric genes^48^ or amplifications of discrete chromosomal regions resulting in the overexpression of certain genes. In addition, other less complex activating (oncogene) or inactivating (tumour suppressor) point mutations may also be present, which may hold prognostic and/or predictive (therapeutic) significance themselves, for example, c-*KIT* mutations in gastrointestinal stromal tumours.^49^ The second more common group of STSs are characterised by more complex, unstable karyotypes. These tumours usually contain multiple genetic alterations that may include unbalanced translocations, chromosome rearrangements (following fusions and/or breakages), whole chromosomal duplications or deletions or aneuploidy. Significant copy number changes resulting in tumour suppressor loss or oncogene amplification may be present, and again other less complex activating or inactivating point mutations may also be found.

In addition to the somatic alterations outlined above, there is a growing recognition of the importance of germline genetic variants in STS development. This has been confirmed by recent analyses, which have shown that 17% of sarcoma patients have a recognised hereditary cancer syndrome, while 10% come from a family with a strong history of cancer and 55% carry pathogenic germline genetic variants.^50^

To combat the difficulties associated with basing STS diagnosis on morphology alone (and the accompanying risk of misdiagnoses), several molecular techniques including immunohistochemistry and fluorescence in situ hybridisation can be employed to identify STS subtype-specific chimeric genes or overexpressed proteins. Examples of assays used regularly in a clinical setting target the overexpressed proteins STAT6 (solitary fibrous tumours) and MDM2 (dedifferentiated liposarcoma), and the fusion genes *EWSR1-FLI1* (Ewing's sarcoma^51^), *FUS-DDIT3* (myxoid/round cell liposarcoma^52^) and *SS18-SSX1/2* (synovial sarcoma^53^). Unfortunately, although these tools are a major advance (particularly in tumours with anon-descript or monomorphic morphology), the small number of genetic alterations that are known to be specific to individual STS subtypes means that a large proportion of high-grade STSs remain unclassified, and are simply termed ‘undifferentiated pleomorphic sarcomas’. This highlights the significant challenges that continue to surround STS diagnosis,^54^ and the importance of ongoing work to identify more clinically useful tumour-specific markers.

Multiple techniques have been used to identify telomerase activity including the TRAP assay, hTERT mRNA detection and the detection of telomerase-synthesised DNA.^55^ ALT activity has also been identified using several techniques including immunofluorescence to detect APBs,^36^ Southern blot analysis to identify heterogeneous telomere lengths^28^ and rolling circle amplification to identify elevated levels of extrachromosomal C-rich circular DNA (the C-circle assay).^34^ Although the presence of either telomerase or ALT is not specific enough to help diagnose specific STS subtypes, these techniques may allow TMMs to be used as a more general marker of malignancy, and to differentiate STSs from other intermediate soft tissue tumours. One caveat to this approach must be the presence of telomerase in several normal cell types^56–58^ and some benign tumours.^59^ To address this issue, the genomic drivers of pathological TMM activity could instead be targeted as markers of malignancy. Examples include *TERT* promoter mutations (an approach already proposed in urological^60^ and thyroid malignancies^61^) and inactivating mutations in *ATRX* and *DAXX* (two genes that when disabled facilitate activation of ALT in several cancers including STSs^43^ ^62^). Although this approach sounds appealing, it should be noted that only 45% of ATRX-deficient STSs appear to be ALT positive, while only 55% of ALT-positive tumour are *ATRX* deficient.^63^ Furthermore, certain malignant tumours also show no evidence of either telomerase or ALT activity, which in some series number up to 50% of STSs.^16^ These cases suggest that current assays are either not sensitive enough to detect telomerase or ALT in these tumours, or that a yet undiscovered TMM is activated. Both of these scenarios raise concerns surrounding sensitivity and specificity, and highlight the need for further research in this area.

### Biomarker development

STSs' aggressive biological behaviour means that following treatment up to 17%^64^ and 24% of cases^65^ recur locally or distantly (with metastases). These are poor prognostic indicators with 5-year survival rates following local and distant recurrence being just 50% and 15%, respectively.^66^ At present, there are no known circulating biomarkers of STS tissue. In their absence, patients with STS are currently followed-up by serial clinical examination accompanied by chest radiographs, and appropriate cross-sectional radiological imaging as indicated. An unfortunate consequence of this approach is that recurrent disease is often too extensive to facilitate curative treatment when diagnosed, which leaves many patients with palliative options alone which as discussed below are extremely limited. All malignant cells need to maintain their telomere length to avoid senescence, achieve replicative immortality and progress. This makes TMM-associated markers an attractive potential source for biomarkers of malignant tissue, especially in a group of cancers as heterogeneous as STSs. Despite this, although C-circles and *TERT* RNA have been proposed as potential markers in osteosarcoma^34^ and carcinoma, respectively,^67^ there is a great paucity of work investigating telomere-associated biomarkers in STSs.

Circulating cancer biomarkers overcome the need for access to tumour tissue by being readily obtainable from the blood. Furthermore, they can easily be accessed repeatedly throughout a course of treatment or follow-up, allowing for serial assessments to be made. In recent years, the hunt for novel circulating cancer biomarkers has led to much interest in the field of circulating cell free DNA (cfDNA). cfDNA is defined as nucleic acids secreted into the circulation. It is clear that patients with cancer have higher levels of cfDNA than healthy individuals due to increased levels of cellular apoptosis and necrosis.^68^ ^69^ In these patients, a proportion of cfDNA (known as circulating tumour-derived DNA (ctDNA)) is shed directly into the circulation from tumour tissue. Although many circulating molecules hold the potential to act as cancer biomarkers, it is the characteristics of this ctDNA that appear to respond most sensitively, and over the widest dynamic range to changes in tumour stage.^70^ ^71^ As a result, ctDNA is a particularly attractive potential tool to monitor disease behaviour and burden, especially as many of the genomic alterations associated with pathological telomere maintenance may theoretically be detectable in ctDNA.

### Therapeutics

The curative treatment of STSs generally involves surgical resection combined with preoperative or postoperative radiotherapy.^72^ Current systemic chemotherapy agents hold no curative role for STSs, with only 35% of patients showing a clinical response when the most commonly used anthracycline-based regimes are instigated for palliation in metastatic cases.^73–75^

A significant proportion of patients with STS present with regional or metastatic disease at diagnosis.^76^ As a result, developing more effective systemic therapies is pivotal to improving outcome for patients with STS. Although several potential therapeutic targets have been proposed,^77^ only a few alternative agents have emerged to the chemotherapeutic regimes currently in use, for example, tyrosine kinase inhibitors in cases of dermatofibrosarcoma protuberans containing platelet-derived growth factor B mutations^78^ and gastrointestinal stromal tumours.^79^ Deepening our understanding of the molecular basis of STS tumourigenesis is key to addressing this issue, and developing new targeted agents effective at treating micrometastatic or radiologically detectable STS deposits.

The need for cancer cells to maintain telomere length to achieve immortality makes inhibiting TMMs an attractive proposition when considering novel therapeutics. Such inhibitors could work in several ways. Successful telomerase function requires transcription and assembly of the various telomerase enzyme components, followed by migration and docking of the enzyme to the telomere undergoing lengthening. Each of these stages is a potential target for antitelomerase agents^80^ with other options including disruption or inhibition of telomerase's catalytic components, or a vaccination-based approach. ALT most likely lengthens telomeres by a homologous recombination (HR)-dependent mechanism. Although complete inhibition of DNA repair by HR would be incompatible with survival, as our understanding of ALT develops pathways specific to tumoural telomeric DNA recombination may be discovered and targeted.

Several issues surround potential systemic cancer therapies designed to target TMMs. First, as discussed earlier a proportion of malignant tumours show no evidence of telomerase or ALT activity using current laboratory assays. Second, the existence of more than one known TMM means that blocking either telomerase or ALT in isolation may prove ineffective. This is particularly concerning as ALT and telomerase activity have been identified simultaneously in both cell lines^81^ and sarcomas.^82^ Third, as telomeres shorten relatively slowly in the absence of an active TMM, malignant cells may continue to survive for a significant period even after telomerase or ALT inactivation, during which time metastatic or regional progression may occur. Finally, the presence of telomerase in several healthy human cell types means the potential side effects of telomerase inactivation must be recognised. These can be predicted from the clinical syndromes associated with shortened telomeres, which includes bone marrow insufficiency and pulmonary fibrosis.^83^

To date, we are aware of three TMM-associated agents that have entered clinical trials—the telomerase-targeting lipid-conjugated oligonucleotide imetelstat (Geron) and the TERT-directed vaccines GV1001 (GemVax, KAEL-GemVax) and GRNVAC1 (Geron vaccine). Imetelstat competitively binds with hTR, blocking telomeric DNA binding. The agent has shown variable results in multiple cancers including osteosarcoma and Ewing's sarcoma,^84^ ^85^ although side effects including neutropenia and thrombocytopenia have been reported. GV1001 injections contain a peptide derived from TERT that stimulates telomerase-specific cytotoxic (CD8^+^) and helper (CD4^+^) T cells to target and kill tumour cells expressing TERT at the cell surface when injected.^86^ GRNVAC1 injections contain antigen-presenting cells activated ex vivo by exposure to hTERT mRNA, which also target TERT-expressing cells.^87^ Both vaccines appear to be well tolerated in current trials, although no sustained significant improvements in patient outcome have been reported to date.^86^ ^88–90^

## Summary

STSs are a rare group of complex tumours with a poor outcome compared with many other solid malignancies. Key to improving patient prognosis is the development of sensitive biomarkers to allow clinicians to detect disease recurrence earlier, and effective systemic therapies to treat regional and metastatic disease. Telomere maintenance provides a common mechanism that characterises all malignant cells. As a result, TMMs should be viewed as attractive avenues to explore as we move into a time when the molecular characterisation of STSs is becoming key to furthering treatment.

**Take home messages**

- Soft tissue sarcomas (STSs) are a rare group of complex malignant tumours with a relatively poor prognosis compared to many other solid cancers.
- Despite optimal treatment a significant proportion of STSs recur locally or at a distant anatomical site. This is a problem magnified by a lack of sensitive STS biomarkers or systemic STS treatments.
- Pathological Telomere Maintenance Mechanisms (TMMs) must be employed by all malignant cells to avoid replicative senescence. As a result TMM markers provide an exciting potential source for novel STS biomarkers, or potential targets for new systemic STS agents.
